# Angiographic and clinical comparison of novel Orsiro Hybrid sirolimus-eluting stents and Resolute Integrity zotarolimus-eluting stents in all-comers with coronary artery disease (ORIENT trial): study protocol for a randomized controlled trial

**DOI:** 10.1186/1745-6215-14-398

**Published:** 2013-11-20

**Authors:** Joo Myung Lee, Sang-Don Park, Sang Yup Lim, Joon-Hyung Doh, Jin Man Cho, Ki-Seok Kim, Jang-Whan Bae, Woo-Young Chung, Tae-Jin Youn

**Affiliations:** 1 Seoul National University Hospital, Seoul, Korea; 2 Inha University Hospital, Incheon, Korea; 3 Korea University Ansan Hospital, Ansan, Korea; 4 Inje University Ilsan Paik Hospital, Goyang, Korea; 5 Kyung Hee University Hospital at Gangdong, Seoul, Korea; 6 Jeju National University Hospital, Jeju, Korea; 7 Chungbuk National University Hospital, Cheongju, Korea; 8 Boramae Medical Center, Seoul, Korea; 9 Division of Cardiology, Department of Internal Medicine, College of Medicine, Seoul National University and Cardiovascular Center, Seoul National University Bundang Hospital, 166 Gumi-ro, Bundang-gu, Seongnam-si, Gyeonggi-do 463-707, Republic of Korea

**Keywords:** Drug-eluting stent, Orsiro hybrid stent, Zotarolimus-eluting stent, Coronary heart disease

## Abstract

**Background:**

The Orsiro Hybrid sirolimus-eluting stent is a newly developed third-generation drug-eluting stent, featuring a unique dual-polymer mix. An active bioabsorbable polymer delivers the anti-proliferative drug, sirolimus, via controlled release, while a passive biocompatible polymeric coating shields the metallic strut from surrounding tissue, preventing interaction. To date, the Orsiro Hybrid sirolimus-eluting stent has excelled in terms of late lumen loss at 9 months in a first-in-man single-arm trial. However, the efficacy and safety data for Orsiro Hybrid sirolimus-eluting stents in a broader population of all-comers are limited. The present study offers an angiographic and clinical comparison of the Orsiro Hybrid sirolimus-eluting stent and the Resolute Integrity zotarolimus-eluting stent in the treatment of patients with coronary artery disease.

**Methods/design:**

The ORIENT trial is a multicenter, randomized, open-label, parallel-arm study designed to demonstrate the non-inferiority of the Orsiro Hybrid sirolimus-eluting stent relative to the Resolute Integrity zotarolimus-eluting stent. A total of 375 patients with a spectrum of coronary artery disease will undergo prospective, random assignment to a Orsiro Hybrid sirolimus-eluting stent or Resolute Integrity zotarolimus-eluting stent (2:1 ratio), for a primary endpoint of in-stent late lumen loss at 9 months by quantitative coronary angiography. Secondary 12-month clinical endpoints are death, target lesion revascularization, target vessel revascularization, myocardial infarction, stent thrombosis and target lesion failure (a composite of cardiac death, target lesion revascularization and target vessel-related myocardial infarction).

**Discussion:**

The ORIENT trial is the first study to date comparing the Orsiro Hybrid sirolimus-eluting stent with the Resolute Integrity zotarolimus-eluting stent for efficacy and safety in a population of all-comers with coronary artery disease.

**Trial registration:**

Clinicaltrials.gov NCT01826552

## Background

Since the introduction of the first-generation drug-eluting stent (DES), rates of restenosis after percutaneous coronary intervention (PCI) have declined dramatically [1-3]. However, there are concerns that the benefit of a DES in reducing restenosis is at the expense of thrombogenic risk. Hence, efforts to reduce both restenosis and thrombosis are ongoing and have resulted in a flurry of second-generation devices made with biocompatible but non-absorbable polymers, as well as third-generation models incorporating bioabsorbable polymers [4,5].

The recently launched Orsiro Hybrid sirolimus-eluting stent (Orsiro SES, Biotronik AG, Bulach, Switzerland) features a unique hybrid polymer laminate over thin cobalt chromium struts. The active BIOlute® bioabsorbable polymer matrix is embedded with the anti-proliferative drug sirolimus and releases the drug in a controlled manner after implantation. This component degrades over time, leaving behind only the PROBIO® encapsulated stent. The passive PROBIO® coating confers a protective interface that guards against a reaction between the stent’s metal framework and the surrounding tissues [6]. Although the Orsiro SES has excelled in terms of late lumen loss at 9 months in a first-in-man single-arm trial (BIOFLOW-I trial) [7], randomized controlled trials evaluating its efficacy and safety are limited to date.

The ORIENT Trial will be an angiographic and clinical comparison of the Orsiro SES, an innovative third-generation DES, with the Resolute Integrity zotarolimus-eluting stent (ZES-I, Medtronic Cardiovascular, Santa Rosa, CA, USA), the latest second-generation DES, in the treatment of patients with coronary artery disease (CAD).

## Methods/design

### Study design

**Figure 1:**
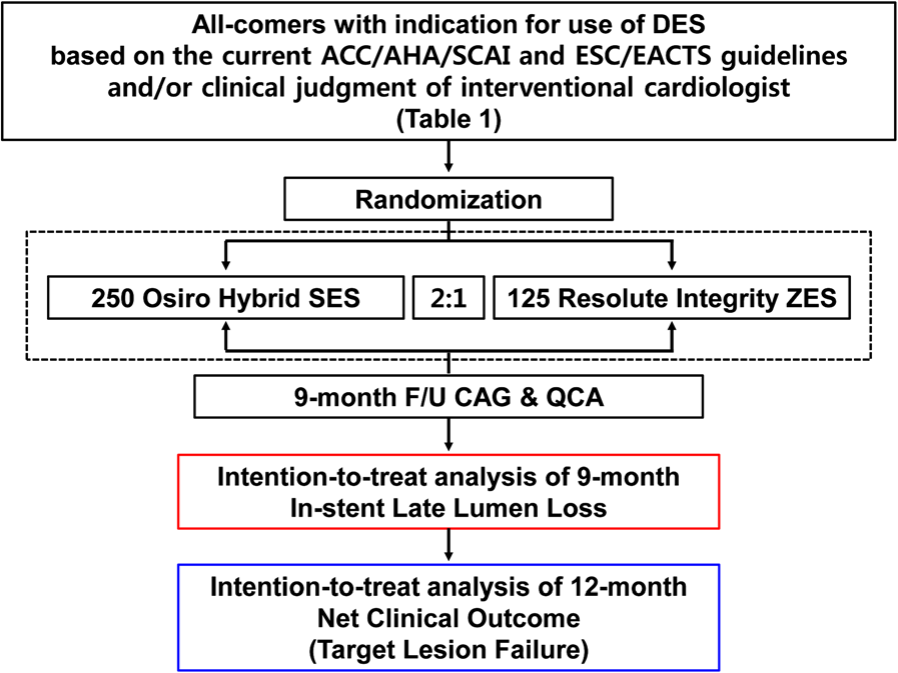
**ORIENT trial algorithm.** ACC, American College of Cardiology; AHA, American Heart Association; CAG, coronary angiography; DES, drug-eluting stent; ESC, European Society of Cardiology; EACTS, European Association for Cardio-Thoracic Surgery; F/U, follow-up; QCA, quantitative coronary angiography; SCAI, The Society for Cardiovascular Angiography and Interventions; SES, sirolimus-eluting stent; ZES, zotarolimus-eluting stent.

This is a prospective, randomized, open-label, and parallel-group multicenter trial designed to test the non-inferiority of the Orsiro SES relative to the ZES-I in preventing late lumen loss 9 months after the index procedure (Figure 1). A total of 375 patients exhibiting a wide spectrum of CAD will be enrolled at eight centers in Korea. Participants will be monitored clinically at months 1, 6, 9 and 12. Investigators may conduct this follow-up as telephone interviews or office visits. An angiographic follow-up to determine late lumen loss is advised at 9 months post-procedure or earlier should the patient experience ischemic symptoms or show non-invasive evidence of ischemia.

This trial is investigator-initiated, with grant support from the Biotronik Korea Co, Korea. Other than financial sponsorship, the company has no role in protocol development or the implementation, management, data collection and analysis of this study. The authors alone are responsible for the design and execution of the trial, related statistical analyses and all aspects of manuscript preparation, including drafting, editing and final content. This study will be conducted according to the principles outlined in the Declaration of Helsinki. All patients must provide written informed consent. This study protocol has been approved by the institutional review board of Seoul National University Bundang Hospital and registered at http://www.clinicaltrials.gov (NCT01826552).

### Study population and entry criteria

Prospective subjects are those at least 18 years of age, presenting with symptomatic CAD (including acute coronary syndrome) and coronary lesions >50%, in whom PCI with DES implantation is indicated, based on the current recommendations of ACC/AHA/SCAI and ESC/EACTS guidelines or the clinical judgment of the interventional cardiologist (Table 1) [8,9]. Whether the PCI is indicated based on current recommendations or not will be separately recorded. Inclusion and exclusion criteria will be graded to minimize exclusion of patients, thus reflecting the population at large. All participants will be randomly assigned in a 2:1 ratio to either the Orsiro SES or ZES-I group. To generate comparable groups relative to known and unknown risk factors, randomization will be independently conducted online via a web-based application (T&W Software, Seoul, Korea). The randomization will be balanced and stratified by participating center and allocated treatment groups.

**Table 1:** Eligibility criteria for the ORIENT trial

**Inclusion criteria**	● Patient age ≥18 years
	● Ability to acknowledge verbally the risks, benefits and treatment ramifications in receiving the Orsiro Hybrid® or Resolute Integrity® stent
	● Written informed consent given by legally authorized agent prior to any study-related procedure
	● Indication for use of drug-eluting stent based on ACC/AHA/SCAI and ESC/EACTS guidelines and/or clinical judgment of interventional cardiologist.
	● Target lesion(s) in coronary artery or graft vessel with estimated reference diameter ≥2.5 mm and ≤5.0 mm
	● Target lesion(s) amenable to percutaneous coronary intervention
**Exclusion criteria**	● Known hypersensitivity or contraindication to any of the following agents: heparin, aspirin, clopidogrel, sirolimus, zotarolimus, cobalt chromium or contrast media^a^
	● Inability to tolerate aspirin or clopidogrel for 1-year duration of study
	● Systemic (intravenous) use of sirolimus or zotarolimus within 12 months
	● Females with childbearing potential (unless negative by a recent pregnancy test) or anticipating pregnancy following study enrollment
	● History of bleeding diathesis, known coagulopathy (including heparin-induced thrombocytopenia), or refusal of blood transfusions
	● Gastrointestinal or genitourinary bleeding within prior 3 months, or major surgery within 2 months
	● Planned major non-cardiac surgery within designated study period
	● Cardiogenic shock (Killip class IV)
	● Symptomatic heart failure, precluding coronary angiography in a supine position
	● Non-cardiac co-morbid conditions limiting life expectancy (to <1 year) or potentially undermining protocol compliance (as judged by the site investigator)
	● Active participation in another drug- or device-related investigational study where the primary endpoint follow-up is ongoing
	● Unwillingness or inability to comply with protocol procedures

^a^Patients with documented sensitivity (such as a rash) to contrast media that is amenable to premedication with steroids and diphenhydramine may still be enrolled. However, true anaphylaxis on prior exposure to contrast media is grounds for exclusion.

### Outcome measurements and definitions

The primary endpoint of the trial is in-stent late lumen loss at 9 months, as measured by quantitative coronary angiography (QCA). Secondary angiographic endpoints at 9 months are in-segment late lumen loss, percentage diameter stenosis (%DS) and binary restenosis. Secondary clinical endpoints include all-cause and cardiac deaths, clinically driven target lesion revascularization (TLR), target vessel revascularization (TVR), myocardial infarction (MI) (target or non-target vessel-related), stent thrombosis and target lesion failure (TLF, a composite of cardiac death, TLR and target vessel-related MI) at 12 months [10,11]. Revascularization is considered clinically driven if the follow-up angiographic diameter stenosis ≥50%, and there is at least one of the following: (1) history of recurrent angina pectoris, presumably related to the target vessel, (2) objective signs of ischemia at rest or during exercise testing (or equivalent), presumably due to the target vessel, (3) abnormal invasive functional diagnostic testing or (4) TLR or TVR with ≥70% diameter stenosis, even without any of the aforementioned ischemic signs/symptoms [10,12].

To reduce the chance of the so-called oculostenotic reflex, whereby rates of repeated revascularization are disproportionately high, investigators will be encouraged to perform a non-invasive stress test or invasive functional diagnostic test (for example, fractional flow reserve) prior to a decision regarding repeated revascularization and they will adhere strictly to a clinically driven revascularization protocol. In addition, the specified indication of revascularization and the number of TVRs or TLRs that are performed without a non-invasive stress test will be separately recorded.

### Coronary angiography and percutaneous coronary intervention

Coronary interventions will be performed according to current standard techniques. Aside from random stent assignment, any pre-dilatation, post-stenting adjunctive balloon inflation, and intravascular ultrasound or glycoprotein IIb/IIIa inhibitor use will be at the operators' discretion. All patients will receive aspirin (300 mg) and a loading dose of clopidogrel (300 mg to 600 mg) prior to the procedure, unless these are routinely taken, for at least 1 week beforehand. Heparin sufficient to sustain activated clotting time at >250 sec will be administered intravenously in boluses during the procedure. All patients will be given aspirin indefinitely (at least 100 mg/day) and clopidogrel (75 mg/day) for a minimum of 12 months after the index procedure.

### Quantitative coronary angiography

The coronary angiography at baseline, immediately after the procedure and at follow-up will be analyzed using an automated edge-detection system (Cardiovascular Angiography Analysis System 5.7.1; Pie Medical Imaging Systems, Maastricht, the Netherlands) at the core laboratory in Seoul National University Bundang Hospital by an experienced technician who is blinded to the study. The minimal lumen diameter (MLD), reference vessel diameter, %DS, acute gain (difference in MLD pre- and post-implantation of the stent), late lumen loss (difference in post-procedural and follow-up MLD) and binary restenosis (≥50% stenosis at the follow-up angiography) will be measured [13]. All QCA assessments of target lesions will be in-stent and in-segment (stent plus 5-mm proximal and distal margins).

### Statistical considerations

#### Sample size calculation

To test the primary hypothesis (non-inferiority of the Orsiro SES relative to the ZES-I by 9-month in-stent late lumen loss), we assumed a mean late loss of 0.3 mm (SD 0.54 mm) for both stents, which was extrapolated from the QCA results for the Endeavor Resolute® (ZES-R) from the RESOLUTE US trial [14]. The ZES-I and ZES-R devices use same the delivery drug (zotarolimus) and basic design, namely a biocompatible polymer (BioLinx®) mounted on a cobalt chromium platform. For non-inferiority testing, with a 0.2 mm non-inferiority margin, type I error at 0.05 (one-sided), 90% statistical power, 2:1 sampling ratio (Orsiro SES:ZES-I) and an expected 30% dropout rate (at the 9-month follow-up angiography), a total of 375 patients (Orsiro SES, 250; ZES-I, 125) were deemed necessary to execute this study. To avoid interlesion clustering of restenosis in patients receiving stents for multiple lesions, which would have required correction with multilevel generalized estimating equations, only one randomly selected lesion will be used in the analysis of in-stent late lumen loss [15,16]. Sequential superiority testing will be performed if non-inferiority for in-stent late lumen loss is met. This number of patients would also have 92% power to detect superiority with an in-stent late lumen loss difference of 0.2 mm between the groups at a two-sided alpha level of 0.05.

#### Statistical analysis

All primary and secondary endpoints will first be analyzed on an intention-to-treat basis (all patients according to the assigned treatment groups), and secondarily, per protocol/treatment. The per protocol analysis is aimed at patients completing the study protocol as initially assigned, without significant breach or crossover, whereas the per treatment analysis addresses the treatment modality received, regardless of initial device assignment. Per protocol/treatment analyses are intended solely for descriptive comparisons, not as tests of the working hypothesis. For the intention-to-treat analysis, all patients giving written informed consent and randomly assigned for the study will be used in the analysis sample, whether or not the treatment rendered was correct or crossover occurred. The endpoints will also be subjected to per patient and per lesion analyses as feasible.

The null hypothesis will be evaluated on a non-inferiority basis, using an independent sample t-test of the mean late lumen loss in both groups. Non-inferiority is defined by a one-sided alpha of 5% and a difference in late lumen loss ≤0.2 mm. TLF, TLR, TVR, MI and stent thrombosis rates at 12 months will be analyzed with a χ2-test, generating Kaplan–Meier survival estimates for time to events of TLF, TLR, TVR and MI at 12 months. Acute success (device, lesion and procedure) analysis will utilize a χ2-test as specified. An independent t-test or χ2-test will be invoked as needed to assess in-stent/in-segment %DS and in-stent/in-segment binary restenosis at 9 months. In addition, exploratory subgroup analysis will be performed, rather than pre-specified subgroup analysis.

### Study organization and ethical consideration

The principal investigator, the study coordinator, and the Clinical Trials Center at Seoul National University Bundang Hospital are jointly responsible for all aspects of the study protocol and amendments. Site monitoring and data collection will be performed by a dedicated affiliated research coordinator. At appropriate intervals, designated trial monitors will review all investigational data for accuracy and completeness to ensure protocol compliance. In addition to the executive committee, the steering committee, data safety and monitoring board and the clinical event adjudication committee will be involved for the duration of the trial.

## Discussion

The first-generation of DESs, armed with sirolimus or paclitaxel, was shown to reduce in-stent neointimal hyperplasia, reduce rates of clinical restenosis and curtail the need for repeated PCI, compared with bare metal stents [17,18]. However, the greater risk of late stent thrombosis raised safety concerns [17]. A number of mechanisms were thought responsible for the thrombotic proclivity of DES-treated arterial segments. In particular, the suboptimal biocompatibility of polymers coating the bulky stainless steel alloy of first-generation devices was held culpable for delayed endothelialization and hypersensitivity reactions [19]. DES-related research and development subsequently embraced long-term safety as well as performance enhancement.

Second-generation devices were thus developed using breakthrough technology, such as thinner stent struts, permanent but biocompatible polymers to minimize inflammation or hypersensitivity reactions, and novel anti-proliferative agents. The ZES family and the everolimus-eluting stent (EES) are second-generation DES prototypes. They are fabricated with cobalt chromium alloy, which imparts superior radial strength and radiopacity. The thinner struts dampen the neointimal response and promote more rapid re-endothelialization [19]. The biocompatible polymers, when coupled with newer anti-proliferative agents, likewise improved drug delivery, reducing neointimal proliferation and causing fewer thrombotic events than older devices [4,5].

In contrast, third-generation devices now incorporate biocompatible polymers, which gradually degrade after implantation. The potential for a late inflammatory reaction due to a non-absorbable polymer is thus avoided, and the subsequent risk of late or very late stent thrombosis is reduced [20]. The Orsiro SES features a hybrid of active and passive polymers, which is unique in its class. Its active BIOlute® coating is made of poly-L-lactide, a well-characterized bioabsorbable polymer, which gives a controlled drug release. The sirolimus drug load is 1.4 μg/mm^2^ and 50% of the drug is released within 30 days and 80% within 3 months. The passive PROBIO® polymer seals the stent surface, reducing the interaction between the metal framework and surrounding tissue and forming a diffusion barrier for blood. Inherent biocompatibility is thereby improved, which reduces thrombogenicity and facilitates re-endothelialization [6]. In addition to advanced encapsulation, the Orsiro SES has a thinner cobalt chromium-based strut (60 μm) than its DES forerunners (ZES-R, 91 μm; EES, 81 μm). Even with the added polymer coating, the total strut thickness is only 71 μm (for a 3.0-mm stent), which is significantly thinner than comparators (ZES-R, 99 μm; EES, 95 μm). The Orsiro SES has shown promise in terms of late lumen loss at 9 months in a first-in-man single-arm trial, and the BIOFLOW-II trial, comparing 9-month late lumen loss of the Orsiro SES with EES, is ongoing [7]. However, a comparison of the Orsiro SES with ZES-I, the latest second-generation DES, has not been done to date. We expect this study to provide valuable data on the therapeutic outcomes of second- and third-generation DES devices.

## Trial status

The ORIENT trial has begun patient randomization in October 2013. The principal investigator may be contacted by email.

## Abbreviations

%DS: Percentage diameter stenosis; CAD: Coronary artery disease; CAG: Coronary angiography; DES: Drug-eluting stent; EES: Everolimus-eluting stent; F/U: Follow-up; MI: Myocardial infarction; MLD: Minimum lumen diameter; PCI: Percutaneous coronary intervention; QCA: Quantitative coronary angiography; SES: Sirolimus-eluting stent; TLR: Target lesion revascularization; TVR: Target vessel revascularization; ZES-I: Resolute integrity zotarolimus-eluting stent.

## Competing interests

The authors declare that they have no competing interests.

## Authors’ contributions

JML contributed to the study design and to drafting and revising the manuscript. TJY, as a principal investigator, provided the study concept and reviewed the manuscript. SDP, SYL, JHD, JMC, KSK, JWB and WYC actively contributed to the study design, and all authors read and approved the final manuscript.
